# Unraveling the gut microbiota-brain axis: Mechanisms, pathophysiology, and therapeutic opportunities

**DOI:** 10.1016/j.isci.2026.116224

**Published:** 2026-06-04

**Authors:** Changsheng Jiang, Wedad M. Nageeb, Shumaila Batool, Amro Hashish, Sabah M. Alkhawagah, Hoda Atef Abdelsattar Ibrahim, Wenlong Zhu, Chuanyan Che, Xiaojin Li, Mengmeng Jin, Xiaoqian Zhang, Ahmed H. Ghonaim, Man Ren, Shenghe Li

**Affiliations:** 1 Anhui Provincial Key Laboratory of Animal Nutritional Regulation and Health, College of Animal Science, Anhui Science and Technology University, Chuzhou 233100, China; 2 Medical Microbiology and Immunology Department, Faculty of Medicine, Suez Canal University, Ismailia, Egypt; 3 Department of Zoology, Wildlife and Fisheries, University of Agriculture Faisalabad 38000, Pakistan; 4 Department of Veterinary Diagnostic and Production Animal Medicine, College of Veterinary Medicine, Iowa State University, Ames, IA, USA; 5 National Laboratory for Veterinary Quality Control on Poultry Production, Animal Health Research Institute, Agriculture Research Center, Giza, Egypt; 6 Medical Microbiology and Immunology Department, Faculty of Medicine (for Girls), Al-Azhar University, Cairo, Egypt; 7 Pediatric Clinical Nutrition Division, Department of Pediatrics, Faculty of Medicine, Cairo University, Giza, Egypt; 8 National Key Laboratory of Agricultural Microbiology, College of Animal Sciences and Veterinary Medicine, Huazhong Agricultural University, Wuhan 430070, China; 9 China Institute of Veterinary Drug Control, Beijing 102629, China; 10 Desert Research Center, Cairo 11435, Egypt

**Keywords:** Psychiatry, Precision medicine, Neuroscience, Immunology, Microbiology, Systems biology

## Abstract

The gut microbiota-brain axis constitutes a dynamic, bidirectional communication network that integrates neural, endocrine, immune, and metabolic pathways to regulate host physiology and behavior. Accumulating evidence indicates that disturbances within this axis have been consistently associated with metabolic, autoimmune, and neuropsychiatric disorders; however, much of the current evidence, particularly from human studies, remains largely correlative, and causal relationships are still under active investigation, highlighting its systemic relevance to health and disease. This review synthesizes current understanding of the structural and functional components of the gut microbiota-brain axis, including microbial community dynamics, neural signaling pathways, and key endocrine and immune mediators. We examine mechanistic insights into how microbial-derived metabolites influence brain function, cognition, mood regulation, stress responses, and disease pathogenesis. In addition, we discuss emerging therapeutic strategies targeting the gut microbiota-brain axis, including psychobiotics and fecal microbiota transplantation, while acknowledging dietary approaches, such as prebiotic supplementation, fiber-rich diets, and fermented food consumption, as complementary strategies that modulate microbiota composition that may indirectly support gut-brain axis function. By integrating mechanistic, clinical, and translational perspectives, this review aims to clarify current knowledge gaps and highlight future directions for leveraging the gut microbiota-brain axis in personalized approaches to neuropsychiatric disease management.

## Graphical abstract

**Figure:**
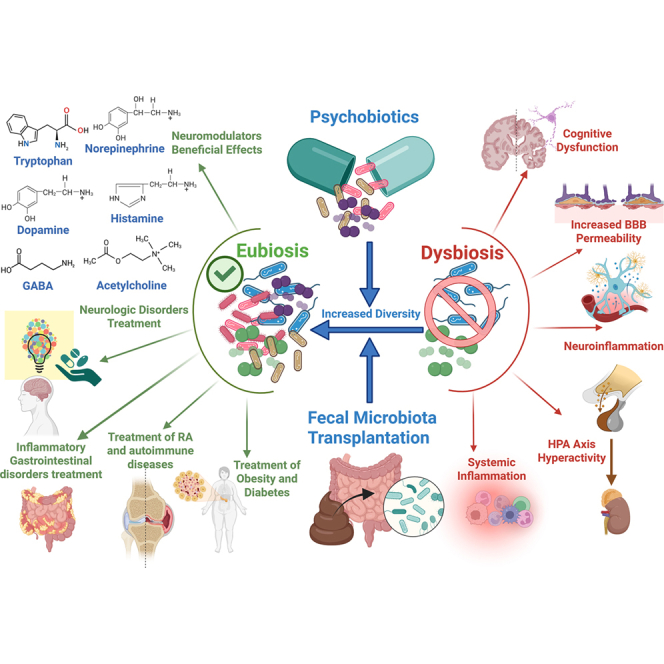

## Introduction

The gut microbiota-brain axis is now recognized as a highly integrated, bidirectional communication network linking the intestinal microbial ecosystem with the central nervous system (CNS) through neural, endocrine, immune, and metabolic pathways.^1^ This axis orchestrates a wide range of physiological functions, including gastrointestinal (GI) motility, secretion, energy homeostasis, stress responsiveness, and higher-order cognitive and behavioral processes.^2^

Mechanistically, gut-brain communication is mediated through multiple interconnected routes, including the vagus nerve (VN), the enteric nervous system (ENS), the hypothalamic-pituitary-adrenal (HPA) axis, immune signaling cascades, and microbial-derived metabolites such as short-chain fatty acids (SCFAs), neurotransmitter precursors, and bile acid derivatives.^3^ Disruption of these finely tuned interactions has been suggested to be associated with the pathogenesis of a broad spectrum of disorders, encompassing neuropsychiatric and neurodevelopmental conditions, neurodegenerative diseases, metabolic and GI disorders, as well as systemic autoimmune diseases.^4^ Animal studies have provided experimental evidence connecting behavioral modulation via neural and metabolic pathways, whereas human studies are mostly observational, showing correlations between microbiota composition and psychological and mental outcomes.^5^ These findings have shifted the gut microbiota-brain axis from a descriptive concept to a central framework for understanding disease mechanisms across multiple organ systems.

Despite the rapid expansion of literature in this field, critical challenges remain. Many existing reviews focus on isolated components of the gut microbiota-brain axis or emphasize associative findings without fully integrating mechanistic, clinical, and translational perspectives within a unified framework. Moreover, inconsistencies in study design, population heterogeneity, and outcome measures have limited the clinical translation of microbiota-based interventions. Most existing reviews either describe mechanistic pathways without systematically evaluating clinical evidence quality, or report clinical findings without grounding them in the mechanistic architecture of the axis. As a result, there remains a substantial gap between experimental insights and their incorporation into evidence-based therapeutic strategies. Importantly, most evidence in human studies remains associative rather than causal, and is influenced by inter-individual variability and methodological differences, which complicate interpretation and limit reproducibility.

This review addresses these gaps through several key contributions that set it apart from existing literature. First, it brings together all four major gut-brain communication pathways, neural, endocrine, immune, and metabolic, into a single, integrated framework. Rather than treating these pathways separately, we focus on how they interact, with particular attention to the molecular specificity of microbial metabolites. These include SCFAs, highlighting their roles in histone deacetylase (HDAC) inhibition and brain-derived neurotrophic factor (BDNF)-related neuroepigenetic regulation, as well as tryptophan-derived metabolites and bile acid species with distinct blood-brain barrier (BBB) permeability and CNS receptor targets. Second, the clinical evidence is organized using a clear hierarchy of study design, ranging from meta-analyses and systematic reviews of randomized controlled trials (RCTs), to individual RCTs, observational studies, and preclinical animal research. This structure is applied consistently across all disease categories, allowing readers to readily distinguish between well-supported findings and those that remain preliminary or associative. Such a transparent, stratified approach is rarely implemented with this level of consistency in narrative reviews. Third, this review incorporates the most up-to-date human evidence, including recent meta-analyses and RCTs across neuropsychiatric conditions (such as depression and autism spectrum disorder [ASD]), neurodegenerative diseases (including Parkinson’s disease [PD], Alzheimer’s disease [AD], and multiple sclerosis [MS]), and GI disorders. Where available, landmark studies published up to 2025 are included to provide a current and comprehensive perspective. Fourth, psychobiotics and fecal microbiota transplantation (FMT) are evaluated from a translational standpoint rather than purely descriptively. We assess not only their reported effects, but also their underlying mechanisms, the strength and limitations of current evidence, and their future potential. In particular, emerging concepts such as precision psychobiotics and genetically engineered microbial strains are discussed as promising directions for the field.

Within this integrated framework, we examine how the structural and functional components of the gut microbiota-brain axis interact to influence brain function and behavior. Emphasis is placed on the pathways linking microbial signals to neural, endocrine, and immune systems, and how these interactions shape cognition, mood regulation, stress responses, and disease susceptibility. Throughout, we carefully distinguish between findings derived from experimental models and those supported by human data. Overall, this review provides a unified and critically evaluated perspective on the role of the gut microbiota in brain health. At the same time, it highlights key gaps in current knowledge and outlines research priorities needed to translate microbiome science into effective neuropsychiatric therapies.

## Key components of the gut microbiota-brain axis and mechanisms of communication

### Gut microbiota

Trillions of microorganisms inhabit the human body, collectively termed “human microbiota.”^6^ While the skin, airways, urogenital tract, and GI tract are major sites of colonization by different microbes, the majority ofthese microorganisms reside in the gut and are referred to as the gut microbiota.^7^ The gut microbiota includes a diverse array of bacteria, viruses, fungi, and archaea that play critical roles in maintaining human health.^8^^,^^9^ Key functions include food fermentation, pathogen resistance, immune modulation, and vitamin synthesis.^10^

**Figure 1:**
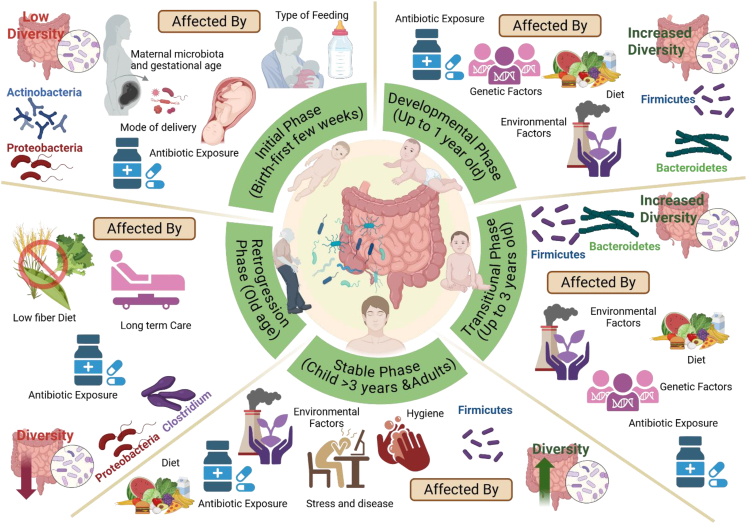
Gut microbiota development across the human lifespan The first few years of life are critical for the establishment of gut microbiota, which can be broadly divided into four phases before reaching stability. Each phase is influenced by multiple factors and is dominated by specific taxa. years of stability, the composition is gradually altered by aging, leading to a decline in diversity and function.

Traditionally, the fetal GI tract was believed to be sterile, with microbial colonization beginning only at birth. However, this paradigm has been challenged by studies detecting microorganisms in the placenta, amniotic fluid,^11^ umbilical cord, and meconium^12^ of healthy newborns, without signs of inflammation or infection. A proposed explanation is that fetuses may swallow amniotic fluid containing microbes, initiating gut colonization *in utero*. This hypothesis is supported by similarities in microbial composition between meconium, and amniotic fluid.^11^^,^^13^ Notably, meconium displays a distinct microbial profile compared to later fecal samples, highlighting dynamic shifts in microbial composition during fetal and early postnatal development.^14^ The first few years of life are essential for the development of gut microbiota and can be broadly divided into four distinct phases, as shown in Figure 1.^13^^,^^15^

#### Phase I (initial phase)

This phase begins immediately after birth and is influenced by several factors including maternal microbiota, gestational age, mode of delivery, diet, and antibiotic exposure.^16^^,^^17^ Early colonizers include facultative anaerobes, such as *E*. *coli*, *Staphylococcus* spp., *Enterococcus* spp., and α-hemolytic streptococci. Oxygen-rich neonatal GI environment inhibits obligate anaerobes. During this phase, microbial diversity is low and predominantly composed of the Proteobacteria and Actinobacteria phyla, with large inter-individual variation.

#### Phase II (development phase)

Starting in the early weeks of life, this phase is heavily influenced by breastfeeding, which significantly shapes microbial composition.^18^ Facultative anaerobes deplete oxygen, creating an anaerobic environment conducive to obligate anaerobes. Human milk oligosaccharides promote the growth of *Bacteroides*, *Bifidobacterium*, and *Clostridium* species.^16^^,^^19^ This phase witnesses an increase in microbial diversity, dominated by the Bacteroidetes and Firmicutes phyla.^19^^,^^20^

#### Phase III (transitional phase)

Occurring after the first year of life, infants develop a distinct microbiota signature that increasingly resembles that of adults.^19^

#### Phase IV (stable phase)

Typically achieved between 2 and 3 years of age, this phase marks the stabilization of gut microbiota into an adult-like community, composed of Firmicutes, Bacteroidetes, and Actinobacteria and dominated by Firmicutes.^19^ Later in life, age-related changes in diet and immune function can shift the microbial composition. For instance, elderly individuals often exhibit reduced *Bifidobacterium* and increased *Clostridium* and *Proteobacteria*. The decline in *Bifidobacterium*, a key anaerobe with immunomodulatory properties, has been linked to heightened inflammatory states in older adults.^10^

Generally, the adult gut microbiota contains over 2,776 bacterial species classified into 11 phyla. More than 90% are represented by Firmicutes, Bacteroidetes, Actinobacteria, and Proteobacteria, while Verrucomicrobia and Fusobacteria are among the least abundant.^10^^,^^21^ The Firmicutes phylum includes over 200 genera such as *Clostridium*, *Bacillus*, *Lactobacillus*, *Enterococcus*, and *Ruminicoccus*, with *Clostridium* accounting for the majority of this group. Dominant genera in the *Bacteroidetes* phylum include *Prevotella* and *Bacteroides*. The Actinobacteria phylum is largely represented by *Bifidobacterium*, while the *Proteobacteria* phylum is chiefly represented by the *Enterobacteriaceae* family.^22^

The gut microbiota composition is highly individualized and influenced by age, medications, and environmental exposures. Microbial diversity also varies along different regions of the GI tract, primarily due to variations in pH, oxygen levels, and nutrient availability.^10^^,^^22^ For instance, the small intestine, with its shorter transit time and higher bile salt concentration, favors the growth of Proteobacteria such as *Enterobacteriaceae*, whereas the colon, characterized by slower transit and a more neutral pH, hosts larger, more diverse communities dominated by anaerobes like *Bacteroidaceae* and *Prevotellaceae*.^10^^,^^23^

Gut microbiota play a critical role in neurodevelopment through the production and modulation of neurotransmitters and neuroactive metabolites, including SCFAs such as propionate and butyrate, which influence microglia maturation, neurogenesis, and neuroinflammation (discussed in detail in short-chain fatty acids). Dysbiosis has been linked to neuroinflammation associated with neurodevelopmental disorders including ASD and schizophrenia. Prenatal and postnatal microbial exposure shapes brain development, with mode of delivery and breastfeeding influencing microbiome seeding and, consequently, cognitive and behavioral outcomes.^24^ Gut microbiota also regulate the HPA axis stress response, a pathway described mechanistically in endocrine pathway, with commensal bacteria serving as key determinants of its developmental trajectory.^24^

Regarding mood, approximately 90% of peripheral serotonin is synthesized in the gut from tryptophan, a process regulated by the gut microbiota, the full mechanistic basis of this relationship is described in tryptophan and its metabolites. Additionally, *Lactobacilli* and *Bifidobacteria* produce GABA, the brain’s primary inhibitory neurotransmitter, linking gut dysbiosis to mood and anxiety disorders. Gut microbes communicate with the brain through three principal routes, neural (neural pathway of gut-brain communication), endocrine (endocrine pathway), and immune/circulatory pathways (immune pathway), which are each examined in detail below.^25^

### Neural pathway of gut-brain communication

**Figure 2:**
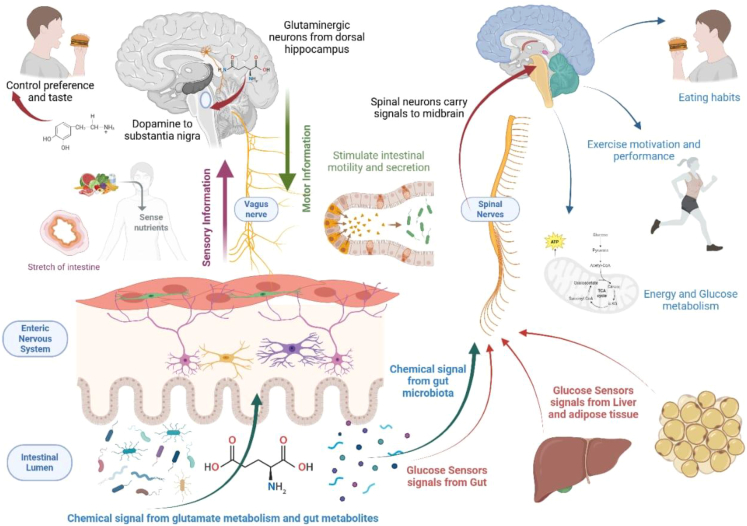
A diagram illustrating the neural pathways of gut-brain communication, which utilizes the vagus and spinal nerves for bidirectional communication between gut and brain

A neural pathway consists of interconnected neurons that transmit signals between the central and peripheral nervous systems, enabling bidirectional communication between the GI tract and the brain.^26^ There are several ways for the gut and brain to communicate with each other, and a major one is the neural pathway, as illustrated in Figure 2.^26^ This system includes the VN, ENS, and spinal nerves. With these nerves, the brain and gut can connect and help control digestion, emotions, immune actions and overall health.

The ENS is responsible for starting the connection between the gut microbiota and the brain.^27^ It includes neurons and glial cells found in the submucosa and muscularis propria of the intestines.^28^ The ENS can control GI activity by itself without the help of the CNS. It may also transmit signals to the CNS via the VN, metabolites produced by microbes and neuroendocrine routes.^29^^,^^30^ Chemicals discharged by gut microorganisms and cells can activate afferent neurons in the ENS, which in turn affect electrical signals in the brain through the VN. Because the nervous system is closely linked to the microbiota, the ENS interacts with the microbiota on a long-lasting and two-way basis. Microbiota finds a suitable environment in the ENS, which contributes significantly to the healthy growth, normal development and structure of the ENS.^31^ Gut microbiota also promotes maturity in the adult ENS by producing 5-hydroxytryptamine (5-HT) and engaging the 5-HT4 receptors. This role of gut bacteria causes alterations in the structure of the ENS and promotes faster movement of food through the gut.^32^

The VN acts as the significant nerve link between the gut and the brain. It uses sensory and motor fibers to allow communication in both directions.^33^ Sensory fibers bring messages from the gut to the brain about feeling full, nutrients present and any uncomfortable sensations in the GI tract.^34^ The brain uses motor fibers to control GI processes, including motility and gastric secretion.^34^ The brain and gut can continuously regulate and provide input because of this bidirectional connection.

Gut microbiota release chemical messages that are delivered to the CNS through the VN.^35^^,^^36^ In most cases, intestinal glutamate metabolism delivers gut luminal information from VN to glutamatergic neurons in the dorsal hippocampus in milliseconds, enabling the topographical resolution and temporal precision of a synapse to sense gut inputs.^37^^,^^38^ Optical stimulation of sensory neurons in the right vagal ganglion of the intestine can cause glutamatergic neurons to release dopamine in the substantia nigra. As a result, brain reward neurons are stimulated and controls preference and taste.^39^ Neurons in the vagal ganglion can also detect stretching in the intestine, control intestinal villi, notice nutrients and manage how intestinal muscles contract. These types of hormonal responses influence both the pattern of eating and the regulation of glucose in the body.

Spinal afferent neurons, which are primary sensory neurons, play a key role in the somatosensory system by relaying sensory signals from the GI tract, liver, adipose tissue, and other tissues to the CNS. Glucose sensors in the intestine and hepatic portal vein stimulate the spinal afferents, which carry this information to the brain.^40^ If this pathway is disrupted by diseases like inflammation or diabetic neuropathy, it can disturb both messaging between the gut and brain and lead to troublesome eating habits, also affecting energy and glucose metabolism.^40^ Studies have demonstrated that by transferring gut microbiota signals to the midbrain, sensory neurons can influence exercise motivation and performance.^17^^,^^41^

### Endocrine pathway

The endocrine system uses hormones to send messages between the gut and brain. HPA axis is an endocrine pathway that connects the brain with the gut. The HPA axis helps control the body’s reaction to many types of stimuli and stresses.^42^ The hypothalamus’ paraventricular nucleus releases corticotropin-releasing factor (CRF) into the body to control the parasympathetic, sympathetic, immune, metabolic, and CNS systems. The two-way signaling between the brain and gut via the HPA axis is known to be essential for overall health. Recent research demonstrated that the endocrine pathway influences the connection among the gut, microbes and the brain in infancy and that the gut microbiome affects the growth of the HPA axis.^43^ Probiotic therapy can control gut bacterial composition, minimize excessive HPA axis activity, and reduce anxiety-related stress response.^44^ When an imbalance in gut bacteria is detected, it activates the pituitary gland and triggers a further amplification of the process. This leads to the release of adrenocorticotropic hormone, which stimulate the adrenal glands to produce cortisol (CORT). It causes a negative cycle involving the pituitary, hypothalamus, and hippocampus.^45^ When there is inflammation or shifts in gut bacteria, it can lead to activation of the HPA axis and cause higher levels of stress hormones such as CORT. These hormones actively participate in several body functions such as mood, thinking and behavior. Too much CRF produced by the hypothalamus is believed to be the underlying cause of abnormal HPA axis function. Being overactive can result in depression and anxiety.^46^ When CORT secretion is impaired, there are issues with the adrenocorticotropic hormone’s response to CRF, the daily cycle of the HPA axis, levels of glucocorticoid and also some brain areas.

Neurotransmitters secreted by the gut are sensed by the brain of the host. The CNS and bacteria in the gut are able to share messages through neurotransmission, assisting with the successful adaptation of microbiota in the GI system.^47^^,^^48^ Most of the cells responsible for making neurotransmitters and hormones in the gut are enteroendocrine cells. Analysis of single-cell transcription at different time points revealed five key enteroendocrine hormones; neurotensin or cholecystokinin, 5-HT, gastric inhibitory peptide, glucagon-like peptide 1 or somatostatin, and ghrelin. These cells show an exciting ability to adjust their hormone production which is constantly changing. With maturity, gut epithelial cells start producing several hormones.^49^^,^^50^ In order to generate neurotransmitter substances such as DA, serotonin and kynurenine, the gut depends on tryptophan and applies both the 5-HT and kynurenine pathways.^51^ Almost all peripheral 5-HT is produced by enterochromaffin cells (ECs), under the influence of gut microorganisms providing 5-HT to the lumen, mucosa, and the blood circulation. Thus, there is increased interaction between the intestine and the CNS.^52^^,^^53^ 5-HT contributes to regulating mood, eating habits, sleep patterns, and various other body functions. After entering the bloodstream, 5-HT in the gut is carried to different CNS parts, affecting the functions of the hypothalamus, cerebral cortex and amygdala.^54^

Additionally, 5-HT interacts with immune cells and gut epithelial cells to influence physiological functions, including bowel movements and intestinal barrier (IB) permeability.^55^ Tyrosine usually creates neurotransmitters such as dopamine, epinephrine, and norepinephrine in the GI tract. Recent researchers have demonstrated that the gut microbiota is able to change tyrosine into 4-ethylphenyl sulfate (4EPS). When this compound gets into the brain, it may stop myelinated oligodendrocytes from maturing, which increases anxiety. Even though 4EPS is classified as a neuroactive substance rather than a typical neurotransmitter, it still influences brain activity and complex animal behaviors.^56^ The memory deficit, cognitive alteration, and depressive behavior could be associated with gut microbiota metabolites and neurotransmitters.^57^^,^^58^ Similarly, other neurotransmitters like GABA and DA also play a role in the brain-gut interaction through affecting behavioral, cognitive, and emotional functions.

### Immune pathway

**Figure 3:**
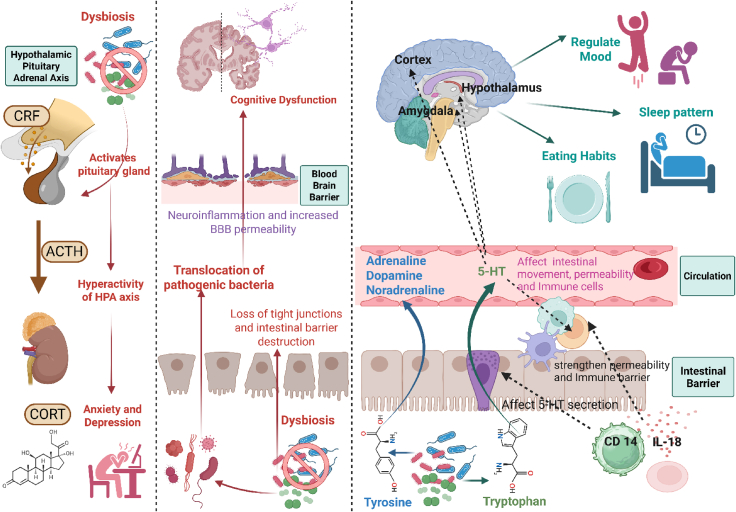
A diagram illustrating neuroendocrine and immunologic pathways of gut-brain communication; the immune pathway, where gut microbiota influences immune responses and maintains homeostasis; and the endocrine pathway, involving gut-derived hormones that regulate systemic metabolic processes CRF, corticotropin-releasing factor; ACTH, adrenocorticotropic hormone; CORT, cortisol; HPA, hypothalamic pituitary adrenal axis; 5-HT, 5-hydroxytryptamine.

Gut immune processes protect the ENS providing a way of interaction between neural pathways and gut microbiota, as depicited in Figure 3.^59^ Macrophages have shown to support the ENS differentiation supporting normal neural function both during healthy infancy and adulthood.^60^^,^^61^ Macrophages were shown to convert to protective phenotypes preventing motility disorders and ENS neuronal loss following infection of gut. The ENS also affects the activity of the gut immunity mediated by related immune responses. It has also been demonstrated that the gut immune barrier can be regulated by the neurons in the gut via the signals produced by interleukin-18 (IL-18), therefore, having a significant impact on the mucosal barrier and on eradication of the invasive bacteria.^62^ It was found that the “gut microbiota-immune barrier homeostasis” could protect the brain against inflammation-related damages in a CD14-dependent process. The homeostasis of the gut microbiota-immune barrier also plays a significant role in the secretion of 5-HT by chromaffin cells within the intestinal epithelium.^63^ This 5-HT has the ability to modulate the gut-brain communication in the peripheral circulation that affects visceral pain and emotions in organisms.^64^ Mechanistically, IL-18 secreted by gut epithelial neurons acts on intestinal epithelial cells to tighten barrier function via caspase-1 activation, illustrating a direct neuroimmune regulatory loop in which ENS signaling maintains mucosal homeostasis and limits bacterial translocation.^62^

A key mechanistic link between gut dysbiosis and neuroinflammation involves the sequential disruption of two barrier systems: the IB and the BBB.^65^^,^^66^ Under homeostatic conditions, tight junction proteins, including claudins, occludins, and zona occludens, maintain IB integrity, restricting microbial translocation. Dysbiosis reduces SCFA production (particularly butyrate, which transcriptionally supports tight junction gene expression via HDAC inhibition), leading to downregulation of these proteins, increased IB permeability, and translocation of microbial products, principally lipopolysaccharide (LPS), into the portal and systemic circulation.^67^^,^^68^ Circulating LPS activates Toll-like receptor 4 (TLR4) on peripheral monocytes and macrophages, triggering NF-κB-mediated release of pro-inflammatory cytokines including IL-1β, IL-6, and TNF-α. These cytokines act on cerebrovascular endothelial cells, promoting downregulation of BBB tight junction proteins and increasing BBB permeability.^69^^,^^70^ Once the BBB is compromised, peripheral immune cells, including monocyte-derived macrophages, can infiltrate the CNS and differentiate into M1-type microglia, perpetuating neuroinflammation and impairing neuronal function.^71^^,^^72^ Dysbiosis caused IB disruption which leads to LPS translocation causing systemic inflammation affecting BBB permeability eventually causing neuroinflammation. This cascade represents a core mechanistic pathway through which gut microbiota composition influences brain health and psychiatric outcomes.

### Gut microbial metabolites

In the GI tract, the gut microbiota metabolizes dietary components to produce bioactive compounds known as gut microbial metabolites. These metabolites play a central role in the gut microbiota-brain axis, mediating bidirectional communication between the gut and the brain.^73^ Key microbial metabolites, presented in Table 1, that affect brain activity and systemic health include tryptophan metabolites, SCFAs, and bile acid derivatives.^73^^,^^74^

**Table 1:** Neurotransmitters and neuromodulators produced by gut microbiota and their function

Bioactive substance	Producers	Effect	Reference
Short-chain fatty acids (SCFAs)	*Bifidobacterium*, *Roseburia*, *Faecalibacterium* ferment fibers into SCFAs (butyrate-propionate-acetate)	• Acetate crosses BBB to influence mood and appetite. • Propionate reduces anxiety-like behavior. • Butyrate supports BDNF protecting against depression. • Butyrate regulates Treg cells and reduces IL-17 suppressing autoimmunity. • SCFAs strengthen gut barrier integrity preventing endotoxin leakage • SCFAs inhibit NF-κB reducing TNF-α, IL-6	Haghikia et al.^200^;Petakh et al.^6^
Serotonin (5-HT)	*Enterococcus*, *Streptococcus*, *E*. *coli*, *Candida* Produced in the gut from precursor tryptophan which availability is enhanced by lactobacillus	• Low serotonin is linked to anxiety, depression, and obsessive compulsive disorders • Anti-inflammatory effect and low levels lead to increased inflammation via T-17 activation	Yano et al.^53^;O’Mahony et al.^201^
Gamma-aminobutyric acid (GABA)	*Lactobacillus*, *Bifidobacterium*, *Bacteroides*	• The brain main inhibitory neurotransmitter. • GABA inhibits proinflammatory cytokines (IL-6-TNF-α). • Reduces stress response and neuroinflammation. • Low GABA is linked to anxiety, insomnia, and panic disorders.	Strandwitz et al.^202^
Norepinephrine	*E*. *coli and Bacillus*	• Modulates mood, focus, fight or flight response. • Stimulates B2 Adrenergic receptors which suppresses IL-6-TNF-α.	Góralczyk-Bińkowska et al.^203^
Dopamine	*Serratia*, *Bacillus*, *E*. *coli* Affect dopamine synthesis via tyrosine metabolism	• Regulates focus, motivation and pleasure. • Dopamine receptors regulate Treg/Th17 balance. • Low dopamine linked to depression, Parkinson’s disease, and ADHD.	Góralczyk-Bińkowska et al.^203^;Hamamah et al.^100^
Acetylcholine	*Bifidobacterium and Lactobacillus*	• Critical for memory, learning, and muscle control. • Vagus nerve signaling leads to improved cognitive functions. • Suppress NF-κB and TNF-α reducing inflammation. • Low acetylcholine leads to Alzheimer’s and cognitive decline.	Park et al.^101^
Histamine	*Morganella*, *E*. *coli*, *Lactobacillus reuteri*	• Regulates mode, appetite, and wakefulness. • Has both proinflammatory and anti-inflammatory effect. • Excess histamine leads to insomnia, anxiety, and brain fog.	Thomas et al.^204^

#### Short-chain fatty acids

SCFAs are primarily produced in the cecum and colon via bacterial fermentation of dietary fibers.^73^ The main SCFAs include acetate, propionate, and butyrate, which together account for approximately 95% of total SCFAs in the gut.^67^^,^^75^ SCFA concentration varies along the gut, being highest in the cecum, moderate in the proximal colon, and lowest in the distal colon.^67^^,^^76^ Multiple factors influence SCFA synthesis, including the type of substrate (fiber), microbiota composition, colonic pH value, gut transit time, and fermentation location.^77^ While dietary fibers are the principal precursors, certain foods like dairy products also contain SCFAs in high concentrations.^67^^,^^78^

SCFAs play essential roles in regulating immune function, host metabolism, and cell proliferation.^79^ Notably, butyrate serves as an energy source for colonocytes, while propionate and acetate can enter systemic circulation through the portal vein to act on the peripheral tissues and the liver. Although peripheral concentrations are lower, SCFAs act as signaling molecules with wide-ranging effects on host physiology.^74^ The CNS-directed mechanisms of individual SCFAs are distinct and increasingly well characterized. Butyrate, beyond its role as a colonocyte energy substrate, acts as a potent inhibitor of HDACs in the brain, promoting histone acetylation and upregulating expression of BDNF, a key mediator of neuroplasticity and a molecule consistently reduced in depression and neurodegenerative conditions.^73^^,^^74^ Acetate crosses the BBB and is taken up by astrocytes and hypothalamic neurons, where it engages free fatty acid receptors (particularly FFAR3/GPR41) to modulate appetite signaling, energy homeostasis, and mood-related circuitry.^67^^,^^73^ Propionate, while largely metabolized in the liver, also activates FFAR3 on enteroendocrine cells and vagal afferent terminals in the gut wall, stimulating GLP-1 and PYY release and generating ascending satiety signals to the brainstem and hypothalamus without requiring direct CNS entry.^67^^,^^73^ These distinct mechanisms, epigenetic regulation (butyrate), direct neuromodulation (acetate), and indirect vagal signaling (propionate), underscore the importance of maintaining a balanced SCFA profile, as dysbiosis-driven shifts in SCFA composition may differentially impair these complementary gut-brain pathways.

#### Tryptophan and its metabolites

Tryptophan is an essential amino acid required for protein synthesis, growth, and health.^80^^,^^81^ It is released in the small intestine during digestion and then absorbed into the bloodstream, mostly bound to albumin.^80^ In the CNS, tryptophan crosses the BBB and is utilized for the synthesis of serotonin (5-hydroxytryptamine), a key neurotransmitter involved in mood regulation, appetite, sleep, and pain processing.^81^ Interestingly, while over 90% of serotonin is synthesized from tryptophan by ECs in the hindgut, this peripheral serotonin cannot cross the BBB, unlike tryptophan itself. This highlights a functional distinction between central and peripheral serotonin.^82^

In the gut, microbial metabolism of tryptophan yields indole and a range of its derivatives.^83^ Specific gut bacteria are associated with this metabolic conversion, including *Lactobacillus*, *Bifidobacterium*, *Bacteroides*, *Anaerostipes*, *Clostridium*, *Ruminococci*, and *Clostridium sporogenes* convert tryptophan into tryptamine.^84^

Indole derivatives serve multiple biological functions. Indole facilitates glucagon-like peptide-1 secretion, delaying gastric emptying and reducing the appetite. Indole and IPA enhance mucosal barrier integrity and reduce permeability of intestine by activating the pregnane X receptor.^84^ Tryptamine promotes serotonin release from ECs, stimulating gut motility via enteric neurons. Furthermore, indole derivatives act as ligands for the aryl hydrocarbon receptor (AhR), a ligand-activated transcription factor. AhR activation contributes to immune modulation and cytokine release (e.g., IL-6, IL-17, IL-22), thereby maintaining intestinal homeostasis and influencing adaptive immunity.^84^

#### Bile acid metabolites

Bile acids are synthesized in pericentral hepatocytes as a result of cholesterol metabolism, producing primary bile acids such as chenodeoxycholic acid and cholic acid.^85^ Food intake triggers their secretion into the intestinal lumen.^85^^,^^86^ Approximately 95% of bile acids are reabsorbed and returned to the liver through enterohepatic circulation; while the remaining 5% the colon where gut bacteria convert them into secondary bile acids through deconjugation and dehydroxylation. These include ursodeoxycholic acid and tauroursodeoxycholic acid.^87^

Bile acids are not only digestive agents; they also function as signaling molecules, regulating lipid digestion, transport, and their own synthesis via nuclear receptors, including the farnesoid X receptor (FXR), and membrane-bound receptors such as the G protein-coupled bile acid receptor TGR5 (also known as GPBAR1).^85^^,^^87^ Both receptor classes are expressed not only in peripheral tissues but also within the CNS, including in astrocytes, microglia, and hypothalamic neurons, positioning bile acids as potential neuroactive mediators.^85^

The capacity of bile acids to access the CNS is critically dependent on their chemical structure and degree of conjugation. Unconjugated secondary bile acids, particularly deoxycholic acid (DCA) and lithocholic acid (LCA), are more lipophilic and demonstrate comparatively greater ability to traverse the BBB, as their hydrophobic properties facilitate passive diffusion and they are less efficiently excluded by efflux transporters such as P-glycoprotein (ABCB1) and ABCG2 expressed at the BBB.^85^ In contrast, conjugated primary bile acids, including taurocholic acid and glycocholic acid, are more polar and hydrophilic, rendering them largely impermeable to the BBB under physiological conditions; their CNS effects are therefore thought to be mediated predominantly through indirect mechanisms, including FXR-driven regulation of fibroblast growth factor 19 (FGF19) signaling and modulation of vagal afferent pathways.^85^^,^^87^

Among the secondary bile acids with documented neuroactive properties, tauroursodeoxycholic acid (TUDCA) and its unconjugated form ursodeoxycholic acid (UDCA) have received particular attention. TUDCA exerts neuroprotective effects through TGR5 receptor activation in neurons and microglia, suppression of the mitochondrial apoptosis pathway, and reduction of endoplasmic reticulum stress, mechanisms that have been demonstrated in preclinical models of PD and AD.^85^^,^^86^ LCA, the most potent endogenous TGR5 agonist, similarly modulates microglial activation and neuroinflammatory signaling, though its effects are dose-dependent and potentially cytotoxic at elevated concentrations. DCA, by contrast, activates both TGR5 and FXR in the brain; while low concentrations may support anti-inflammatory signaling, elevated DCA, as occurs in dysbiotic states, it has been associated with increased neuroinflammatory burden and disruption of BBB tight junction proteins.^86^^,^^87^

It is important to distinguish between direct CNS effects mediated by bile acids that physically cross the BBB, and indirect neuromodulatory effects mediated through peripheral receptor activation. Gut-derived bile acids activate TGR5 receptors on vagal afferent neurons and enteroendocrine cells in the intestinal mucosa, generating ascending neural signals that influence hypothalamic and brainstem function without requiring systemic or CNS entry.^85^^,^^86^ This indirect route may be particularly relevant for conjugated bile acids that cannot efficiently cross the BBB. Understanding this distinction is essential for interpreting the clinical implications of bile acid dysregulation, as different therapeutic strategies, including FXR agonists, TGR5 modulators, and microbiota-targeted interventions that reshape the secondary bile acid pool, may engage these pathways through distinct mechanisms.

Disruption of bile acid metabolism and signaling is linked to diseases affecting the liver, intestine and kidney.^87^ In addition, bile acid-mediated gut-brain signaling has gained interest due to its implications in several neurological and psychiatric disorders, including PD, cognitive decline, and depression, where altered secondary bile acid profiles, particularly reductions in TUDCA and UDCA, have been reported in patient cohorts, though causal relationships remain to be established.^85^ Collectively, the neural, endocrine, immune, and metabolic pathways described above do not operate in isolation but function as an integrated, interdependent network. For example, microbial SCFAs simultaneously reinforce IB integrity (immune pathway), stimulate vagal afferents (neural pathway), modulate HPA axis activity (endocrine pathway), and regulate energy metabolism (metabolic pathway). Similarly, tryptophan-derived serotonin bridges endocrine signaling with immune modulation and neural transmission. Understanding this cross-pathway integration is essential for interpreting how gut dysbiosis can produce multisystem effects and for designing interventions that target multiple nodes simultaneously.

## Implications of gut microbiota-brain axis in health disorders and diseases

The following section examines the clinical implications of gut microbiota-brain axis dysregulation across three major disease domains: neuropsychiatric and neurodevelopmental disorders, GI disorders, and metabolic and autoimmune conditions. Throughout this section, evidence is presented according to study design hierarchy: systematic reviews and meta-analyses of RCTs represent the strongest clinical evidence; individual RCTs follow; observational, cross-sectional, and cohort studies are identified as providing associative data that cannot establish causality; and findings from animal models are explicitly designated as preclinical. This stratification is essential because the field is characterized by substantial heterogeneity in study design, population, and microbiome assessment methodology, which limits direct comparison across studies and the generalizability of conclusions.

### Mental health and neurological disorders

Having established the mechanistic architecture of the gut microbiota-brain axis, encompassing neural, endocrine, immune, and metabolic pathways, and their integration through microbial metabolites, the following section examines how dysregulation of this axis manifests across three major disease domains: neuropsychiatric and neurodevelopmental disorders, GI disorders, and metabolic and autoimmune conditions.

#### Autism spectrum disorder

ASD encompasses a heterogeneous group of neurodevelopmental conditions defined by severe impairments in social communication and interaction, together with restricted and repetitive behavioral patterns. Individuals with ASD frequently exhibit comorbid symptoms such as heightened anxiety and cognitive dysfunction.^88^ While ASD has traditionally been regarded as a primarily neurological disorder, accumulating evidence from both observational and interventional human studies suggests that the GI system and its microbiota are significantly associated with its pathophysiology and symptomatology, although causal relationships remain to be definitively established.

##### Observational and correlative human evidence

Approximately 70% of children with ASD report GI disturbances, indicating altered gut physiology.^89^ Human cross-sectional studies consistently demonstrate altered gut microbiota profiles in children with ASD compared to neurotypical controls, though no single universally identified microbial signature exists.^90^ Documented alterations include an increased *Firmicutes/Bacteroidetes* ratio, higher abundance of *Collinsella*, *Dorea*, and certain *Lactobacillus* species, and lower levels of *Bacteroides*, *Bifidobacterium*, and *Parabacteroides*.^91^ Increased abundance of the fungal genus *Candida* and positive correlations between *Erysipelotrichaceae* and *Lachnospiraceae* abundance and ASD severity have also been reported in human cohort studies.^91^ A large-scale meta-analysis of 16 human studies involving 777 IBS patients extended the observation that dysbiosis, reflected in elevated *Firmicutes/Bacteroidetes* ratios, aligns with gut-brain axis disruption across functional GI disorders, including in neurodevelopmental contexts. In children with ASD specifically, observational human data confirm altered metabolite profiles, including reduced fecal SCFA concentrations and disrupted tryptophan metabolism, compared to neurotypical controls, though these associations cannot confirm causal directionality.^92^

##### Mechanistic evidence

The mechanisms through which gut dysbiosis may influence neurodevelopment in ASD have been characterized predominantly in preclinical models, with translational evidence in humans remaining limited. Reductions in *Bacteroides* and *Bifidobacterium*, genera involved in GABA synthesis via glutamate fermentation and butyrate production, result in reduced GABAergic signaling and butyrate availability in animal models, with consequences including neuronal hyperexcitability, repetitive behaviors, and anxiety-like phenotypes.^93^^,^^94^^,^^95^ Reduced butyrate in preclinical settings has further been linked to disrupted IB function, altered CNS immune responses, and dysregulated serotonin and dopamine signaling associated with social and behavioral impairments.^96^ While human observational data on metabolite profiles are consistent with these findings, mechanistic evidence of these pathways in human ASD populations requires adequately powered longitudinal studies.

##### Clinical RCT-level and interventional evidence

The human interventional evidence base for ASD has expanded considerably. A 2025 systematic review encompassing 33 clinical studies, including 16 RCTs, 14 non-randomized trials, and three retrospective studies, evaluated probiotics, prebiotics, synbiotics, and FMT across behavioral and GI outcomes.^97^ Probiotics showed moderate improvements in behavioral symptoms, with multi-strain formulations outperforming single-strain preparations. FMT demonstrated the most consistent and sustained improvements in both ASD-related behavioral and GI outcomes across all nine FMT-specific studies included, with adverse events limited to minor, transient GI symptoms.^90^ A separate meta-analysis including 15 RCTs identified across major databases found that gut microbiome-based interventions, including probiotics, prebiotics, dietary changes, and fecal transplants, produced measurable improvements in ASD and comorbid ADHD symptom scores.^89^ An RCT involving 41 children with ASD found that FMT recipients showed notable reductions in CARS, SRS, and ABC scores compared to placebo, though the small sample size and short duration limit interpretation.^92^ A pilot RCT (*n* = 30) reported improvements in working memory, analytical thinking, and sensory responses following 6 months of probiotic therapy, similarly constrained by sample size and incomplete blinding.^98^ A meta-analysis of multi-strain probiotic preparations across seven studies demonstrated statistically significant improvements in behavioral outcomes, whereas single-strain preparations showed no significant effect.^91^^,^^99^ The evidence base is also supported by an open-label microbiota transfer therapy study in 18 children with ASD, which reported approximately 80% reductions in GI symptom scores and sustained behavioral improvements at 8-week follow-up, though open-label design limits interpretation.^89^

##### Evidence limitations

Despite these promising findings, no microbiota-targeted intervention has demonstrated sufficient evidence to support clinical recommendations in ASD at this time. Studies are uniformly constrained by small cohort sizes, heterogeneous diagnostic criteria and outcome measures, lack of standardized microbiome assessment, and inability to establish causal relationships.^100^^,^^101^ Large-scale, double-blind, placebo-controlled RCTs with standardized behavioral outcome measures are required to advance this field toward clinical translation.

#### Depression and anxiety

##### Observational and correlative human evidence

Altered gut microbiota profiles have been consistently documented in individuals with depression and anxiety disorders across multiple large-scale human studies. A gut microbiome-wide association study of 1,054 participants from the Rotterdam study cohort, validated in 1,539 subjects from the Amsterdam HELIUS cohort, identified 13 bacterial taxa significantly associated with depressive symptoms, including *Eggerthella*, *Subdoligranulum*, *Coprococcus*, *Sellimonas*, and *Lachnoclostridium*, genera collectively involved in the synthesis of glutamate, butyrate, serotonin, and GABA.^102^ These associations were replicated across two independent cohorts and remained significant after controlling for lifestyle, medication, and confounding variables, representing among the most methodologically robust human microbiome-depression associations to date.^102^ A separate case-control study of 106 antidepressant-naive patients with major depression and 151 healthy controls demonstrated significantly reduced alpha diversity in depressive patients, with 11 taxa exhibiting differential abundance, including reduced *Dialister* and *Lactococcus* and elevated *Hungatella*, *Sellimonas*, and *Lachnoclostridium*, and 36 altered functional pathways including LPS biosynthesis and amino acid metabolism.^103^ However, these observations are cross-sectional and cannot determine whether dysbiosis precedes or results from mood disorder pathology, nor can they exclude confounding from diet, physical activity, and psychotropic medication use.

##### Preclinical evidence

Animal model studies provide mechanistic support for a contributory role of gut microbiota in mood dysregulation. Transfer of gut microbiota from depressed human patients to germ-free rats has been shown to induce behavioral and physiological features of depression in the recipient animals, suggesting that microbial composition may participate in causal pathways.^104^^,^^105^ These findings are mechanistically informative but cannot be directly extrapolated to humans given fundamental differences in microbiome composition, immune system maturation, and behavioral phenotyping. The HPA axis dysregulation described in endocrine pathway, neurotransmitter pathways described in gut microbial metabolites, and neuroinflammatory cascades through the LPS→TLR4→NF-κB pathway described in immune pathway represent the proposed mechanistic framework through which gut dysbiosis may influence mood in humans, though most evidence supporting these links in mood disorder populations remains associative rather than mechanistic.^106^^,^^107^

##### Clinical RCT-level evidence

The most robust clinical evidence for microbiota-targeted interventions in mood disorders comes from meta-analyses of RCTs. A systematic review and meta-analysis of 23 RCTs (*n* = 1,401 participants with clinically diagnosed depression or anxiety) demonstrated that probiotic supplementation produced significant reductions in depressive symptom scores, with more limited efficacy observed for prebiotics and synbiotics.^108^ A separate meta-analysis of 12 RCTs (*n* = 707 participants) confirmed significant reductions in depressive symptoms, particularly with *Lactobacillus* and *Bifidobacterium* species using the Beck Depression Inventory, though results across rating scales were mixed.^109^ Greater efficacy was observed for multi-strain formulations and adjunctive use alongside antidepressants rather than probiotic monotherapy. For anxiety specifically, meta-analytic data indicate consistent anxiolytic effects of psychobiotics, though effect sizes are generally small to moderate and significant inter-trial heterogeneity limits firm conclusions.^25^ Regarding FMT, a 2025 meta-analysis of 12 RCTs (*n* = 681 participants) demonstrated that FMT significantly reduced depressive symptoms (SMD = −1.21; 95% CI, −1.87 to −0.55; *p* = 0.0003), with sensitivity analysis confirming statistical significance (SMD = −0.56; 95% CI, −0.86 to −0.26; *p* = 0.001), representing an important new tier of clinical evidence for gut microbiota-targeted intervention in depression.^110^ Both oral capsule and direct GI administration routes demonstrated efficacy across the included trials.^110^

##### Evidence limitations

Despite these promising signals, significant methodological limitations persist. Study populations vary substantially in diagnostic criteria, baseline microbiome composition, and concomitant medication use. Probiotic strains, doses, and treatment durations differ widely across trials, limiting pooled analysis. Long-term follow-up data are scarce, and placebo-controlled designs with adequate blinding remain insufficiently standardized. The FMT meta-analysis, while statistically significant, includes substantial clinical heterogeneity across delivery routes, populations, and follow-up durations. Consequently, microbiota-targeted interventions cannot yet be recommended as standalone treatments for depression or anxiety, and further rigorously designed RCTs with standardized protocols are required.^111^

#### Neurodegenerative diseases

##### Observational and associative evidence

Altered gut microbiota composition has been reported in patients with AD, PD, Huntington’s disease, and ALS across multiple cross-sectional and case-control studies.^112^^,^^113^ In PD patients specifically, human studies consistently identify reduced *Prevotella* abundance, increased *Enterobacteriaceae*, and reduced SCFA-producing genera compared to healthy controls. Human observational studies have additionally demonstrated that PD patients exhibit significantly elevated serum and fecal concentrations of toxic secondary bile acids, particularly deoxycholic acid (DCA), alongside reductions in neuroprotective ursodeoxycholic acid (UDCA) and tauroursodeoxycholic acid (TUDCA), patterns that correlate with the degree of gut dysbiosis and have been linked to markers of BBB endothelial injury in human-derived models.^114^ In AD patients, human cross-sectional studies identify reduced beneficial taxa including *Akkermansia*, *Blautia*, and *Clostridium butyricum*, alongside cognitive deficits, while elevated cerebrospinal fluid levels of trimethylamine N-oxide (TMAO), a gut microbiota-derived metabolite linked to altered bile acid metabolism, have been documented in AD and MCI patients and correlate positively with established AD biomarkers including phosphorylated tau and amyloid-β.^115^ In MS, a systematic review of 12 clinical studies identified consistent gut dysbiosis including reduced microbial diversity and decreased SCFA-producing bacteria, though significant heterogeneity in diversity metrics and findings across studies limits firm conclusions.^116^ Human observational studies in RA demonstrate enrichment of *Prevotella copri* in early, treatment-naive RA patients, a finding consistently replicated across US, Chinese, Japanese, and European cohorts, alongside reduced *Faecalibacterium prausnitzii*, with *P*. *copri* abundance correlating with disease activity and declining following disease-modifying drug treatment.^117^^,^^118^ However, for all conditions, these associations are observational; the direction of the microbiome-disease relationship and the extent to which dysbiosis precedes or follows neurodegeneration remain unclear in human populations.

##### Preclinical evidence

Animal model studies provide mechanistic support for a role of gut microbiota in neurodegeneration. Key proposed mechanisms include (1) dysbiosis-associated increases in gut and BBB permeability allowing microbial products and inflammatory mediators to promote neuroinflammation; (2) microbial modulation of protein misfolding pathways, with particular relevance to α-synuclein aggregation in PD, where human-derived microbiota has been shown to promote α-synuclein pathology in germ-free mouse models; and (3) altered production of SCFAs and tryptophan derivatives that modulate glial cell activation and neuronal survival.^113^^,^^119^ These mechanisms are supported primarily by rodent and germ-free animal studies, and their direct translation to human neurodegenerative disease remains to be established.

##### Clinical evidence

Human clinical evidence for microbiota-targeted interventions in neurodegenerative diseases has expanded substantially. In PD, a 2025 meta-analysis of RCTs demonstrated that gut microbiota-targeted therapies, including probiotics, synbiotics, and FMT, produced statistically significant improvements in motor function (MDS-UPDRS III; SMD, −0.34; 95% CI, −0.57 to −0.11; *p* = 0.004), bowel function, and oxidative stress markers^2−^ˢᵘᵖ^119^. A concurrent systematic review of FMT-specific clinical trials (six studies including four RCTs, 104 total patients) further reported post-FMT improvements in UPDRS scores, constipation symptoms, and non-motor symptom scores, with no FMT-related fatalities.^120^ A landmark RCT published in JAMA Neurology (Scheperjans et al., 2024) randomized 45 PD patients (2:1) to intracaecal FMT or placebo, demonstrating stronger donor-dependent microbiota engraftment in the FMT group and a significant improvement in the timed up and go test at 6 months (*p* = 0.04), though no significant changes were seen in UPDRS total scores or constipation at 12 months, reflecting the complex and time-limited nature of FMT effects in this population.^121^ The GUT-PARFECT phase 2 double-blind RCT similarly reported reductions in daily motor OFF time and self-reported non-motor symptoms following extended FMT therapy in PD patients, though effects were not sustained across the full 6-month follow-up.^122^ In AD and mild cognitive impairment (MCI), a systematic review of four RCTs (*n* = 293 participants) evaluating *Lactobacillus* and *Bifidobacterium* supplementation reported beneficial effects on cognitive function, altered gut microbiota composition, and improved metabolic biomarkers.^123^ A 12-week double-blind active-controlled RCT demonstrated that multi-strain probiotic supplementation in AD patients significantly improved BDNF levels, reduced oxidative stress markers, and improved cognitive function scores compared to active control.^115^ A triple-blind RCT enrolling 90 older adults with MCI and AD reported that probiotic supplementation produced significant treatment-group interactions for TNF-α, GDNF, and NGF levels relative to placebo.^124^ In MS, a systematic review of 13 clinical interventional studies, including dietary protocols, probiotic supplementation, FMT, and intermittent fasting, reported that probiotics produced improvements in EDSS disability scores, MS-related fatigue, and inflammatory biomarkers, while FMT showed early clinical improvement signals in a limited number of trials.^125^ No meta-analysis of RCTs has yet been published for probiotic interventions specifically in ALS.

##### Evidence limitations

The neurodegenerative disease literature is constrained by critical gaps. Most human studies are cross-sectional, short-term, and inadequately powered to detect clinically meaningful effects on disease progression. Confounders including age, diet, co-medications, particularly dopaminergic agents in PD, and inter-individual microbiome variability are rarely controlled. The RCT evidence, while growing, is marked by significant heterogeneity in patient selection, probiotic strains, FMT protocols, and outcome measures. Sex differences in microbiome composition may independently influence disease risk and treatment response, underscoring the need for stratified analyses and personalized approaches.^126^^,^^127^ High-quality longitudinal RCTs with standardized microbiome assessment and clinically validated outcome measures are required before microbiota-targeted interventions can be considered for incorporation into neurodegenerative disease management.

### Gastrointestinal disorders

Observational and mechanistic evidence linking gut microbiota dysbiosis to GI disorders, including irritable bowel syndrome (IBS) and inflammatory bowel disease (IBD), is substantial; however, this section stratifies human evidence hierarchically, from large-scale observational data through RCT-level interventional evidence. Both conditions involve disruptions in the bidirectional communication between the gut and the brain, involving neural, hormonal, immune, and microbial pathways.^128^

#### Observational human evidence

IBS is a common functional GI disorder characterized by altered bowel habits, abdominal pain, and bloating without visible structural damage, reflecting dysfunction in gut-brain signaling.^129^^,^^130^^,^^131^ Human observational studies consistently demonstrate gut dysbiosis in IBS patients. A cross-cohort meta-analysis of 9,204 datasets from 14 IBS microbiome discovery cohorts identified consistent bacterial species and functional signatures associated with IBS, including two distinct IBS-enriched microbiota clusters: obligate anaerobes typically found in the gut, and facultative anaerobes typically present in the oral cavity, implicating oral bacterial translocation as a potential contributor to IBS pathogenesis.^129^ A case-control study and cross-cohort analysis of 567 IBS patients and 487 healthy controls demonstrated that gut bacteria are significantly less diverse in IBS, with altered abundance of 21 bacterial species, though causal relationships remain uncertain.^132^ A 2024 human study in constipation-predominant and mixed-type IBS subtypes found an increased *Firmicutes/Bacteroidetes* ratio, elevated *Actinobacteria* and *Verrucomicrobiota*, and reduced *Bacteroidota* compared to healthy controls.^133^ In IBS, psychological stress activates the HPA axis, releasing CORT which affects gut motility and sensitivity, while dysbiotic microbiota independently generates neuroactive metabolites acting on the ENS, illustrating the bidirectional gut-brain nature of IBS pathophysiology.^132^

#### RCT-level interventional evidence

At the highest evidence tier, a network meta-analysis of 67 RCTs, encompassing 54 probiotic trials, seven prebiotic/synbiotic trials, and six FMT trials, demonstrated that probiotics, particularly *Bifidobacterium* and *Lactobacillus* strains, significantly improved IBS symptom scores compared to placebo, while prebiotics and synbiotics did not show significant benefit.^133^ A systematic review and meta-analysis of seven placebo-controlled FMT RCTs found that five of seven trials reported positive results for IBS symptom reduction and gut microbiota modulation, though the optimal delivery route, donor selection, and patient population remain undefined and effect sizes were heterogeneous.^134^ A parallel RCT in IBS patients comorbid with anxiety and depression demonstrated that 12 weeks of oral FMT capsules significantly reduced both IBS severity scores and anxiety/depression scores compared to empty capsule controls, reinforcing the gut-brain-behavior connection in this population.^132^ For antibiotic-based therapy, rifaximin, a minimally absorbed antibiotic targeting small intestinal bacterial overgrowth, has received regulatory approval for diarrhea-predominant IBS based on RCT evidence of symptom reduction, providing further clinical validation of a microbiota-targeted mechanism in IBS.^130^

#### Rotavirus and gut-brain neuroimmune signaling

Evidence from viral gastroenteritis research provides additional insights into gut-brain communication. Cellular alterations in the rotavirus-infected intestine emerge before clinical symptoms and resolve prior to viral clearance, suggesting that diarrhea and vomiting arise through neuroimmune mechanisms rather than direct tissue injury alone.^135^^,^^136^ Proposed mechanisms include impaired villus perfusion, diminished enterocyte absorptive capacity, the viral enterotoxin NSP4, and ENS stimulation.^137^^,^^138^^,^^139^ These findings reinforce that GI symptoms arise from complex neuroimmune and microbial interactions rather than structural pathology.

#### Human evidence

IBD, encompassing ulcerative colitis and Crohn’s disease, is characterized by chronic GI inflammation with clear structural and immune system changes.^140^ Human clinical studies demonstrate that IBD is associated with gut dysbiosis, reduced microbial diversity, expansion of pro-inflammatory taxa, and disrupted IB function, all of which activate immune cascades and drive chronic inflammation.^134^^,^^140^ Notably, in human IBD patients, proinflammatory cytokines including IL-6 and TNF-α, which are significantly elevated, have been demonstrated to cross the BBB and influence mood and behavior, explaining the high prevalence of anxiety and depression observed in IBD cohorts.^134^ A double-blind RCT in patients with active ulcerative colitis (*n* = 36) further demonstrated that sodium butyrate supplementation significantly reduced anxiety and depression scores on the Hospital Anxiety and Depression Scale, alongside reductions in inflammatory markers including erythrocyte sedimentation rate and NLR, providing direct human RCT evidence linking butyrate restoration to both GI and psychological outcomes in IBD.^140^ These findings collectively strengthen the clinical relevance of the gut-brain axis in GI disorders.

### Metabolic and autoimmune conditions

Evidence linking gut microbiota dysbiosis to metabolic and autoimmune conditions is derived from a combination of human observational studies, mechanistic data, and preliminary interventional trials. While causal relationships have not been established in human populations, the breadth and consistency of human observational data across multiple conditions supports the clinical relevance of this axis.

#### Metabolic conditions, human observational and mechanistic evidence

Metabolic disorders including obesity, type 2 diabetes mellitus (T2DM), and metabolic syndrome are consistently associated with gut dysbiosis in human studies.^141^^,^^142^ Key mechanisms linking gut microbiota to metabolic disease, including LPS-driven low-grade systemic inflammation, dysregulated SCFA production, and altered energy extraction, are described in detail in short-chain fatty acids and the immune pathway described in immune pathway.^143^^,^^144^ In human cohort studies, gut microbiota composition predicts future metabolic risk: elevated *Firmicutes/Bacteroidetes* ratios and reduced *Akkermansia muciniphila* abundance have been documented in obese and T2DM patients compared to lean controls. Elevated acetate levels in humans with insulin resistance have been associated with hypothalamic appetite dysregulation and increased food intake, reinforcing bidirectional gut-brain-metabolic interactions.^145^^,^^146^ Causal inference remains limited in human populations, and whether dysbiosis precedes or follows metabolic disease is an active area of longitudinal investigation.

#### Rheumatoid arthritis, human observational evidence

In human RA populations, gut dysbiosis has been one of the most consistently characterized and replicated findings in the autoimmune disease literature. Human studies across US, Chinese, Japanese, Finnish, and European cohorts consistently demonstrate enrichment of *Prevotella copri* in patients with new-onset, treatment-naive RA relative to healthy controls and patients with other inflammatory arthritides, with *P*. *copri* abundance correlating positively with disease activity and declining following initiation of disease-modifying antirheumatic drug therapy.^117^^,^^118^ Gnotobiotic human microbiota transfer studies have further demonstrated that *Prevotella*-dominated microbiota isolated from RA patients contributes to Th17-cell-dependent arthritis in humanized mouse models, while *Prevotella histicola*, a distinct species, suppresses arthritis development, illustrating the importance of species-level distinctions within the same genus.^147^ Additionally, reduced *Faecalibacterium prausnitzii* and *Collinsella* enrichment, the latter linked to increased intestinal permeability, have been reported as consistent human RA microbiome signatures across 16 clinical studies.^148^ A preclinical mechanistic model (K/BxN) characterizes how Th17 differentiation driven by segmented filamentous bacteria colonization drives autoantibody production and joint-targeted inflammation, a pathway proposed to be relevant in human RA through the *P*. *copri*-Th17 axis, though direct mechanistic evidence in humans has not been established.^118^ The VN’s cholinergic anti-inflammatory pathway, HPA axis dysregulation, and serotonin and GABA modulation of immune responses have been proposed as additional gut-brain–immune links in RA flare activity, though human evidence for these specific mechanisms remains indirect.^149^^,^^150^

#### MS, human observational and interventional evidence

In MS, human observational studies across 12 datasets identified consistent gut dysbiosis including reduced *Prevotella* and *Faecalibacterium prausnitzii*, both SCFA producers, alongside enrichment of pro-inflammatory taxa.^116^^,^^151^ A systematic review of 13 MS-specific clinical interventional studies, including dietary protocols, probiotic supplementation, FMT, and intermittent fasting, found that probiotic supplementation produced improvements in EDSS disability scores, MS-related fatigue, and inflammatory biomarkers, while FMT showed early clinical improvement signals in a limited number of trials.^116^ A recently published review of MS microbiome modulation studies identified that dietary interventions, particularly omega-3 PUFA-based formulations, additionally reduced relapse rates and improved metabolic features in RRMS patients in individual RCTs.^152^ Demyelination, reduced oligodendrocyte numbers, axonal loss, and astrocyte activation, key MS pathological features, have been linked in animal models to gut-derived immune activation through disrupted IB integrity, though direct mechanistic evidence in human MS remains to be established.^153^

#### Systemic lupus erythematosus, human evidence

In SLE, human gut microbiome profiling studies document a reduction in the *Firmicutes/Bacteroidetes* ratio and enrichment of *Rhodococcus*, *Eggerthella*, *Klebsiella*, *Prevotella*, and *Flavonifractor* compared to healthy controls, though the pathogenic significance of these specific taxa and their contribution to SLE autoimmune mechanisms remain unclear.^117^^,^^118^

#### Evidence limitations

Across all metabolic and autoimmune conditions reviewed here, human evidence is primarily observational and cannot establish causality. Cross-sectional study designs, small cohorts, inadequate confounder control, and heterogeneous microbiome assessment methods limit interpretation. The question of whether dysbiosis precedes and contributes to disease onset or reflects secondary consequences of disease pathology, inflammation, and immunosuppressive treatment remains unresolved in human populations. Clinical interventional trials targeting the microbiota in RA, MS, SLE, obesity, and T2DM are currently limited in number and methodological quality, and represent a critical priority for future rigorously designed RCTs with standardized microbiome assessment and clinically validated primary endpoints.

## Therapeutic implications

### Role of probiotics in supporting mental health and management of mental health conditions

Probiotics are live bacteria providing health benefits to the human or animal host when administered in sufficient quantities. As per FAO/WHO standards, it must contain viable microorganisms upon consumption and be supplied in an adequate dosage (1 billion to 10 billion CFUs) for efficacy. It must provide clinically proven health benefits and provide strain-specific advantages for the targeted condition. Probiotics strains, listed in Table 2, with mental health benefits are known as psychobiotics. These show promise as an adjunct therapy for mental health by producing and modulating neurotransmitters, reducing inflammation, and improving gut-brain communication. When consumed in adequate amounts, these show measurable improvements in psychological functions including treatment of anxiety, depression, or cognitive improvements. Psychobiotics primarily include *Lactobacillus* and *Bifidobacterium* strains.^154^

**Table 2:** Probiotic strains with reported beneficial effects on mental health from clinical studies

Probiotic strain	Mental health effect	Mechanism of beneficial effect	Reference
*Lactobacillus plantarum PS128*	Beneficial in ADHD, decrease autism behavioral symptoms	Improves dopamine levels, decrease proinflammatory cytokines	Liu et al.^191^
*Lactobacillus plantarum* and *Bifidobacterium breve*	Improve cognitive functions	Improve memory and reduce brain fog.	Zhu et al.^192^
*Lactobacillus rhamnosus* (JB-1)	Reduces anxiety, Decreased stress-related behaviors	Modulates GABA, affects vagus nerve, corticosterone release	Kelly et al.^193^
*Lactobacillus helveticus R0052* and *B*. *longum R0175*	Improve mood and reduce major depressive symptoms	Tryptophan modulation and enhanced serotonin production	Wallace et al.^194^; Kazemi et al.^195^
*Bifidobacterium infantis*	Helps ASD symptoms, irritability, and social behavior	Reduces inflammation	Ishizuka et al.^196^
*Bifidobacterium longum 1714*	Lowers stress perception, improves cognition and memory	–	Allen et al.^197^
*L*. *plantarum DR7*	Decrease anxiety and stress, improves cognitive functions	Decrease proinflammatory cytokines, increased dopamine and norepinephrine	Chong et al.^198^
*L*. *gasseri CP2305*	Improved sleep quality and mental well-being, decreased stress	–	Nishida et al.^199^
*L*. *plantarum JYLP-326*	Improved anxiety, depression, and insomnia symptoms	Changes in fecal metabolites including Cyclohexylamine and Ethyl sulfate	Zhu et al.^192^
*Bifidobacterium breve CCFM1025*	Improvements in depression	Positively impacting the intestinal microbiota and tryptophan metabolism	Tian et al.^44^
*L*. *plantarum HEAL9*	Improvements in depression	–	Saccarello et al.^205^
*Bifidobacterium bifidum (Bf-688)*	Enhance neuropsychological performance, improved symptoms of inattention and hyperactivity in ADHD Improvements in their impulsivity, hyperactivity, and other clinical symptoms	Reduced N-glycan biosynthesis	Wang et al.^206^
*Lactobacillus paracasei (LP-37)*	Reducing perceived stress with stabilizing cortisol levels through controlling HPA, enhances cognition by preventing chronic stress-related deficits in recognition memory and effective in improving cognitive dysfunction, Effective in treating neurodevelopmental disorders in children	Activation of HPA and vagus nerve stimulation, leading to regulation of BDNF and neurotransmitters including Serotonin, GABA, and Dopamine	Allahyari et al.^168^

#### Mechanisms through which psychobiotics affect mental health

Probiotics can positively affect mental health either by helping in producing beneficial metabolites and neurotransmitters or by reducing neuroinflammation. Psychobiotics can additionally affect enteroendocrine hormones and signaling, cytokines and VN.^155^ These metabolites are carried to the circulation, affecting the host’s immune responses. These can also impact local metabolism and neuronal cells, affecting the afferent VN and directly connecting the GIT to the brain. Probiotics strains produce beneficial metabolites such as SCFAs that control host CNS physiology and behavior, improving the cognitive function in animal models. SCFAs like butyrate also support BDNF, which is crucial for neuroplasticity. In that respect, psychobiotics can increase BDNF levels that support cognitive function and neuronal survival, which is also important in the treatment of depressive symptoms.^156^ Probiotic treatment strains like *Bifidobacterium longum* and *Lactobacillus helveticus* also increase the availability of tryptophan, which is a precursor of serotonin that controls several functions, including mood, emotional state, and behavior.^155^

Certain probiotics enhance production of norepinephrine and dopamine, improving motivation, learning, and focus. *Bifidobacterium* and *Lactobacillus* species can produce GABA which promotes relaxation, improves sleep quality and reduces anxiety. *Lactobacillus* species, especially *Lactobacillus rhamnosus* were shown to regulate GABA receptor activity via the VN, decreasing anxiety and depressive symptoms. GABA signaling is also linked to cognitive benefits.^157^

On the other hand, probiotics has a role in strengthening the gut barrier and preventing leaky gut, which consequently reduces systemic inflammation that affects the brain. Some probiotic strains have been shown to reduce proinflammatory cytokines, including TNF-α, IL-1β, IL-6, linked to depression and also to increase the anti-inflammatory cytokine IL-10. The immunoregulatory effects of probiotics have been reported to activate the population of T regulatory cells as well as the secretion of IL-10. Psychobiotics also interact with enteroendocrine cells (EECs) to produce neurotransmitter and neuropeptides such as glucagon-like peptide-1 and -2, peptide YY (PYY), substance P, neuropeptide Y (NPY), serotonin, and cholecystokinin.^158^ Moreover, they interact with gut-associated lymphoid tissue (GALT), to reduce systemic inflammation and modulate immune responses. Probiotics can regulate NK cell activity partly through IFN-γ secretion.^159^ Psychobiotics also reduce microglial activation associated with neuroinflammation and depression. Probiotics can also modulate the HPA axis, reducing CORT levels and improving stress resilience. CORT modulates neuroimmune signaling responses that affect the IB integrity. Some strains can also prevent stress-induced reduction in hippocampal neurogenesis, which is crucial for memory and mood regulation.^160^ Evidence suggests that some psychobiotics may affect HDAC activity, influencing gene expression patterns related to mood and cognition with potential effects on DNA methylation patterns that regulate neurotransmitter synthesis and neural development. Probiotics additionally show promise in schizophrenia treatment through modulating the immune response, reducing inflammation, and potentially improving cognitive function through pathways involving NMDA receptors.^161^

#### Capabilities of different probiotic strains of producing neurotransmitters with clinically reported health benefits

Different bacterial strains demonstrate varying degrees of neurotransmitters production. *Bifidobacterium* and *lactobacillus* species have the ability to synthesize acetylcholine GABA.^162^ Regarding GABA production, *Levilactobacillus brevis* LB01 has been identified as the most efficient probiotic strain for converting monosodium glutamate to GABA, with *Lactiplantibacillus plantarum* 299v also shown as an efficient producer.^163^ Each of *Lactobacillus rhamnosus* JB-1, *Lactobacillus plantarum* 90sk, and *Bifidobacterium adolescentis* 150,^164^ have also demonstrated efficient GABA production.

Regarding serotonin, different bacterial species are either direct producers or modulators of serotonin metabolism. *Enterococcus* and *Streptococcus* families are known as direct producers of serotonin.^25^ *Lactobacillus plantarum* PS128 has been shown to elevate serotonin (5-HT) and dopamine levels in the striatum.^165^ The probiotic species *Lactobacillus* and *Bifidobacterium* have been shown to improve the metabolism of tryptophan by inhibiting the kynurenine pathway, consequently increasing production of serotonin.^166^ Bacillus family members produce both dopamine and noradrenaline.^25^ Various *Lactobacillus* species produce neuroactive substances, including dopamine and serotonin, which may counteract reduced neurotransmitter activity in depression.^166^

#### Clinical evidence about the use of psychobiotics in the management of different mental health conditions

The strongest clinical evidence for psychobiotics in mood disorders has been summarized in depression and anxiety, which reviewed the highest-tier meta-analytic data supporting their use in depression and anxiety.^108^ Building on this evidence base, the following paragraphs focus on the clinical application of psychobiotics across a broader range of mental health conditions, including neurodevelopmental disorders, psychotic disorders, and neurodegenerative diseases. A separate meta-analysis of 12 RCTs (*n* = 707 participants) further confirmed a significant reduction in depressive symptoms, particularly using the Beck Depression Inventory, in favor of probiotic strains including *Lactobacillus* and *Bifidobacterium* species, although results across different rating scales were mixed, underscoring the importance of cautious interpretation.^109^ Notably, greater efficacy was observed for multi-strain formulations and when probiotics were used as adjunctive therapy alongside antidepressants, rather than as stand-alone treatment. Regarding anxiety, meta-analytic data suggest a consistent anxiolytic effect of psychobiotics, though effect sizes are generally small to moderate and significant heterogeneity across trials limits firm conclusions.^25^ A small pilot RCT (*n* = 30 children) reported improvements in working memory, analytical thinking, and sensory responses following 6 months of probiotic therapy in children with ASD.^98^ While these findings are encouraging, the limited sample size, lack of blinding in some assessments, and absence of long-term follow-up constrain generalizability. A double-blind RCT in college students with ADHD (*n* = 60) found that 3 months of multi-strain probiotic supplementation significantly decreased hyperactivity, improved GI symptoms, and enhanced academic performance.^167^ A parallel pilot RCT (*n* = 32 drug-naive children and adolescents) found that *Lactobacillus rhamnosus* GG (LGG) supplementation over 3 months improved health-related quality of life, including social, physical, emotional, and school functioning, though effects on core ADHD symptom scores assessed by parents and teachers were less consistent.^168^ A meta-analysis of five RCTs reported a statistically significant reduction in total PANSS scores following probiotic supplementation as adjunctive therapy (SMD = −0.608; *p* = 0.035). A larger meta-analysis of eight RCTs (*n* = 648 participants) similarly found a significant reduction in total PANSS scores (*p* = 0.03), particularly for negative symptoms.^169^ However, significant heterogeneity was noted across studies (I^2^ = 69%–85%), and effect sizes were small. The limited number of trials and variability in probiotic strains, dosing, and co-supplementation regimens (e.g., vitamin D, selenium) preclude definitive conclusions, and further rigorously designed clinical trials are required.

Systematic reviews have reported promising effects of probiotics on cognitive function, oxidative stress, and inflammation in AD patients, with no reported serious adverse effects.^123^ Literature shows that psychotropics have the potential for use in the treatment of neurodegenerative disease by reducing oxidative stress, inflammation, neuronal aging, and CORT levels and thus increasing levels of antioxidants and neurotransmitters and synaptic plasticity.^170^ Similarly, MS patients receiving probiotic supplementation over 12 weeks showed improvements in the expanded disability status scale and neuropsychological parameters, as well as reduced pain and fatigue in a separate study.^125^ However, these findings are largely based on small, methodologically heterogeneous trials, and more extensive high-quality clinical trials are required before any recommendations can be made. The psychobiotics literature also reports potential benefits in neurodegenerative conditions more broadly, including reductions in oxidative stress, neuroinflammation, CORT levels, and synaptic plasticity impairment, though this evidence base remains primarily observational or derived from early-phase trials.

Emerging evidence supports a potential role for psychobiotics in eating disorders and substance use disorders. Dysbiosis has been proposed to disrupt pathways linked to satiety, reward, impulsivity, and mood, and preliminary data suggest that probiotic supplementation may help regulate food intake and improve satiety-related outcomes, though clinical evidence remains very limited.^171^ Similarly, chronic use of psychostimulants, alcohol, and opioids is associated with gut dysbiosis characterized by a shift toward Bacteroidetes dominance and reduced Firmicutes, which may in turn influence neurobiological reward mechanisms.^172^ Preliminary preclinical evidence suggests probiotics may represent a promising future therapeutic avenue for substance use disorders, but human clinical data are currently insufficient to support any recommendations.^173^

#### Psychbiotics research limitations, concerns, and future directions

Although encouraging evidence currently exists, transition to evidence-based clinical practice is challenged by several limitations. First, current clinical studies lack standardized evaluation protocol and exhibit a high level of heterogeneity. Also, there are significant regulatory concerns in addition to some safety concerns related to the use of live biotherapeutics. No current consensus exists in relation to optimum dosing and formulations due to heterogeneity of probiotic strains and variable dosing regimens used in the available clinical studies. Current research evidence is also hindered by lack of long-term clinical trials which may limit broad generalization of available results. For example, in the treatment of schizophrenia, although promising evidences do exist, some findings related to reduction of positive and negative symptoms are mixed due to diversity of phyla studied which hinder results generalization.^164^ Another challenging aspect for the clinical translation of current research evidence is reproducibility concern that originates from methodologic, environmental or biologic factors. Lack of strain specificity, dosing, and treatment duration variability greatly affect generalization of research findings.^174^ In addition, several modifying factors which are rarely controlled in clinical trials may influence response to psychobiotics such as different dietary habits, physical activity and concurrent medications.^175^ The composition of the gut microbiome in different individuals can influence the response to probiotics. Various strain-specific effects and host characteristics influence the impact of probiotics on various individuals. The dosage and length of use influence efficacy, perhaps augmented when combined with prebiotics (synbiotics) and through the utilization of numerous formulations employing a multi-strain strategy to support production of multiple neurotransmitters. Several methodologic gaps also exist in current clinical trials including small sample sizes, short intervention duration and absent long-term follow-up to assess persistent effect and colonization patterns. There is also a lack of standardized disease progression pattern and lack of homogenous outcomes measures. Although limited, some safety concerns exist with the use of live biotherapeutics especially in vulnerable immunocompromised population such as elderly and hospitalized patients requiring mental health treatments. In these patients with compromised immune system and weak IB, risk of blood stream translocation of probiotic organism has been reported.^176^ Another safety concern originates from the theoretical risk of transferring resistance elements from probiotic strains to other gut commensal genera. Although not clinically reported, this potential risk warrants mandatory screening of all psychobiotic strains for carriage of transferable resistance elements before market approval.

Another challenge exists in the lack of international standards for psychobiotics commercialization where a gap exists in the regulatory classification of psychobiotics as dietary supplements or biotherapeutic products. This leads to unclear regulatory pre-market regulations required to validate clinical efficacy studies.^177^

In order to overcome these limitations, The “Strengthening The Organization and Reporting of Microbiome Studies” (STORMS) checklist has been developed for research transparence to standardize research reporting for clarity and reproducibility of evidence with the aim of facilitating comparative analysis of published results to build robust evidence to develop clinical guidelines.^178^

**Figure 4:**
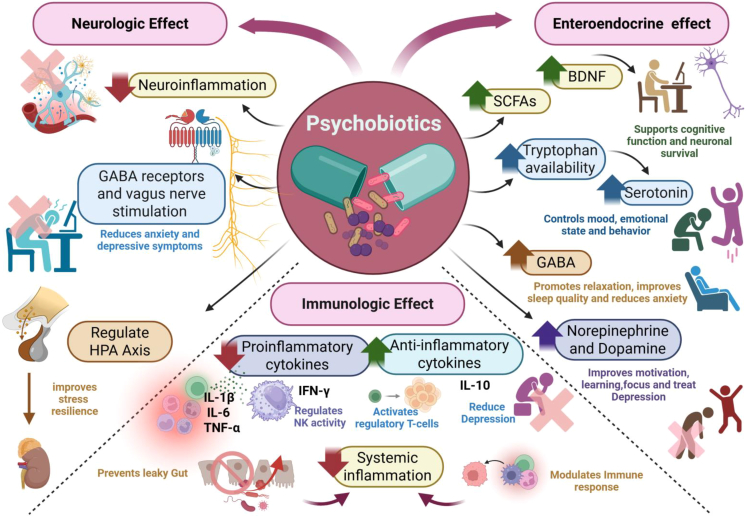
Schematic representation of the mechanisms through which psychobiotics exert beneficial effects on gut-brain axis components Psychobiotics influence the axis through multiple parallel pathways, including production of SCFAs that support IB integrity and modulate CNS physiology; enhancement of BDNF levels supporting neuroplasticity; modulation of the HPA axis stress response; regulation of GALT immune activity; and direct neurotransmitter production influencing vagal afferent signaling.

Future strategies to enhance the efficacy of probiotics in mental illness involve investigating the optimal combinations of strains and selecting customized or tailored strains based on individual microbiome assessments, referred to as personalized psychobiotics, as shown in Figure 4. Also, future research should be targeted to “precision psychobiotics” where patients are stratified based on baseline ecology and predictive biomarkers to help optimize efficacy of treatment and minimizing unwanted risks.^179^ Additionally, genetically engineered probiotics are being tried for targeted neurotransmitter delivery. Combination therapies of probiotics and other medications such as antidepressants/therapy, may also be recommended for better outcomes. Other intriguing avenues also concentrate on interspecies microbiome interactions with a metabolic emphasis. Increased focus is directed to SCFA producers, especially Bacteroidetes and certain Firmicutes species, which indirectly affect neurotransmitter synthesis.^180^

In the upcoming decade, research should prioritize transparency, personalization, and safety by adopting rigorous standards such as STORMS, mandatory probiotic strains screening, and developing a regulatory framework for distinguishing biotherapeutic agents from health promoting dietary supplements.

### Fecal microbiota transplantation and mental diseases

#### Role of fecal microbiota transplants in mental diseases

FMT is a therapeutic intervention that aims to transfer fecal material from a healthy donor into the GI tract of a recipient to reconstruct compromised gut microbiota. Because FMT aims to restore a healthy gut and shows potential promise as a therapy for various gut-related conditions, it was suggested as a treatment approach to mental health conditions through modulation of neurotransmitters levels and reducing inflammatory reactions associated with most mental health conditions. FMT has been associated with improvements in mood and cognitive function in patients with major depressive disorders,^181^ also demonstrating improvement in the quality of life measures.^182^

FMT has shown effectiveness in several experimental and animal studies in the treatment of a wide variety of mental health conditions, including depressive and anxiety-like behavior. Evidence from human studies on FMT in neuropsychiatric disorders remains limited but shows the potential of FMT as a promising therapeutic approach. In clinical cases, very few studies have been performed, indicating that FMT may reduce depressive symptoms when used as an adjunctive therapy in patients previously unresponsive to standard treatment.^183^ While further research is necessary, a single detailed case study reported that FMT was associated with improved mood stability in a patient with bipolar disorder, with a reduction in medication burden observed over the follow-up period.^184^ This finding is anecdotal and derived from a single case report; it cannot be generalized to establish clinical efficacy and should be interpreted with considerable caution pending controlled prospective studies. Additional human studies have also suggested beneficial effects of FMT on anxiety symptoms and self-rated health,^185^ as well as improvements in mental well-being, improving sleep quality, and overall mood.^186^ A systematic review has indicated that FMT consistently resulted in decreased depressive and anxiety-like symptoms across various populations. This body of evidence included clinical studies involving human subjects as well as preclinical studies using animal models.^187^ Current evidence also suggests the potential benefit of FMT in other psychiatric conditions, including schizophrenia and ASDs.^188^ However, these findings remain preliminary and are based on limited, methodologically heterogeneous studies, underscoring the need for cautious interpretation.

FMT is regarded as a generally safe procedure; however, certain safety concerns warrant attention, including the risk of transmitting pathogenic organisms and procedure-related complications, which may range from mild to moderate GI symptoms to more severe adverse outcomes associated with the method of administration.^189^

The long-term consequences of FMT remain under investigation, with the potential for unforeseen or unfavorable outcomes. The enduring efficacy of FMT in alleviating depressive and anxiety symptoms has been documented for several months post-treatment^183^ with certain studies indicating a reduction in the severity of these symptoms lasting up to 6 months. Nonetheless, the long-term effect on neurotransmitter levels and metabolic balance, as well as their correlation with mental health, are currently under investigation. Moreover, other unforeseen long-term consequences resulting from the introduction of novel microbial communities into the gut, such as the potential emergence of immune-mediated illnesses or other health issues, are currently being monitored.^185^

For all these reasons, it is important to develop standardized clinical protocols with personalized donor-selection. Genetic profiling is advised for identifying the optimal donors by determining candidates based on their microbiome makeup and aligning microbiome profiles for potential compatibility with the recipient’s gut flora. Genetic profiling can also be utilized for safety evaluation by screening for genetic markers associated with transmissible illnesses or high-risk organisms.^190^ Personalized treatment approaches should also take into account different environmental, lifestyle and dietary factors for more effective FMT strategies tailored to the individual’s life circumstances. FMT has become a valuable instrument for managing various mental health disorders, with future initiatives focused on conducting additional clinical studies to evaluate long-term safety and efficacy, creating personalized donor selection protocols, implementing continuous monitoring, and formulating ethical guidelines for donor selection and screening procedures.

#### Limitations, regulatory considerations, and safety concerns

Despite their therapeutic promise, both psychobiotics and FMT face significant methodological, regulatory, and safety limitations that constrain clinical translation. Despite recent advances in microbiota-targeted therapies, including psychobiotics and FMT, which have shown promising therapeutic potential, significant concerns regarding safety, regulatory frameworks, and reproducibility, remain. Psychobiotics are generally regarded as safe, particularly in healthy populations, although significant issues remain regarding strain-specific effects, long-term safety, possible interactions with host microbiota, and variability in product formulation.^129^^,^^130^^,^^131^^,^^132^^,^^133^^,^^134^^,^^135^^,^^154^^,^^155^^,^^156^^,^^157^^,^^158^^,^^159^^,^^160^^,^^161^ In addition, the lack of standardized dosing regimens and regional regulatory control complicates reproducibility and restricts the application of research results to normal clinical practice.

Similarly, FMT has established efficacy in recurrent GI infections, but its application in neuropsychiatric disorders remains experimental.^166^^,^^191^^,^^192^^,^^193^^,^^194^^,^^195^^,^^196^^,^^197^^,^^198^^,^^199^ Uncertainties about the long-term safety of FMT and the risk of transmitting infectious agents, especially in vulnerable or immunocompromised individuals, are major concerns. Furthermore, significant variability in donor selection, stool processing, storage, and delivery methods raises issues of reproducibility and standardization. Collectively, these factors underscore the need for well-designed, large-scale RCTs with standardized protocols and long-term safety evaluation to better define the efficacy, safety, and mechanisms of microbiota-based interventions before their widespread clinical application.

### Limitations of current evidence and future research priorities

Despite the significant advances outlined in implications of gut microbiota-brain axis in health disorders and diseases and therapeutic implications, the current evidence on the gut microbiota-brain axis still faces several important limitations that should be carefully considered.

First, much of what we know about microbiome-disease relationships in humans comes from cross-sectional observational studies. While these studies are valuable for identifying associations, they cannot determine whether microbial changes are a cause or a consequence of disease. In addition, factors such as diet, medication use, lifestyle, and natural variability between individuals can strongly influence the microbiome, making it difficult to isolate true disease-related effects. As a result, for many of the conditions discussed in this review, including neuropsychiatric, neurodegenerative, metabolic, and autoimmune disorders, the available human evidence remains largely associative rather than causal. Second, although RCTs investigating microbiota-targeted interventions are increasing, they still present several challenges. Many studies involve relatively small sample sizes and short intervention periods, and there is considerable variability in probiotic strains, dosages, and study designs. Differences in microbiome analysis methods and the lack of long-term follow-up further complicate interpretation. Together, these issues limit the generalizability of findings and make it difficult to draw firm clinical conclusions. Third, while animal studies have provided critical mechanistic insights, translating these findings into human health outcomes remains a major challenge. Differences between species, in microbiome composition, immune system development, and even behavioral responses, mean that results observed in animal models do not always apply directly to humans. This gap is particularly evident in complex conditions such as neuropsychiatric and neurodegenerative disorders. Another important limitation is the lack of standardized outcome measures and reporting frameworks across studies. Without consistent methodologies and definitions, comparing results between studies becomes difficult, slowing progress toward evidence-based clinical guidelines. Finally, for FMT, several key uncertainties remain. These include how donors should be selected, how transplanted microbiota establish and persist in recipients, and what the long-term immunological effects may be. There are also ongoing concerns about safety, particularly the potential transmission of pathogens or antimicrobial resistance genes, which is especially relevant for immunocompromised individuals.

Addressing these challenges will require a more coordinated and systematic research approach. Future studies should focus on large-scale, well-powered, longitudinal RCTs with standardized protocols and clearly defined outcomes. Incorporating mechanistic biomarkers will be essential to better understand how microbiota-targeted interventions exert their effects. In addition, greater attention should be given to factors such as sex and age, which are known to influence microbiome-brain interactions but are often overlooked. Importantly, there is a need for human studies that directly test the mechanisms suggested by preclinical research, including IB function, vagal signaling pathways, and the role of microbial metabolites in neuromodulation. At the same time, the development of clear regulatory frameworks, particularly those distinguishing live biotherapeutic products from dietary supplements, will be critical for advancing clinical applications such as psychobiotics and FMT. Finally, wider adoption of standardized reporting guidelines, such as the STORMS checklist for microbiome studies, will improve transparency and reproducibility. This, in turn, will facilitate more reliable comparisons across studies and accelerate the development of robust, evidence-based clinical recommendations in microbiota-targeted neuropsychiatric medicine.

## Conclusion

The gut microbiota-brain axis has emerged as a fundamental regulator of neurophysiological and behavioral processes, linking the intestinal microbial ecosystem to the CNS through interconnected neural, endocrine, immune, and metabolic pathways. Increasing evidence demonstrates that dysregulation of this axis contributes to the development and progression of neuropsychiatric, neurodegenerative, metabolic, GI, and autoimmune disorders. Despite substantial advances, major challenges remain in translating preclinical discoveries into clinical practice, particularly in establishing causality, accounting for interindividual variability, and determining the long-term efficacy and safety of microbiota-targeted interventions. Therapeutic strategies including psychobiotics and FMT represent the most clinically advanced microbiota-targeted interventions reviewed here; alongside these, dietary approaches, including prebiotic-rich and fiber-diverse diets, fermented food intake, and Mediterranean-style dietary patterns, offer accessible, low-risk strategies for supporting gut microbiota diversity and indirectly modulating gut microbiota-brain axis function. Precision microbiome engineering, including personalized psychobiotics and genetically engineered strains, represents a promising but still early-stage avenue for individualized neuropsychiatric medicine. A deeper understanding of the gut microbiota-brain axis will be critical for transforming mechanistic insights into effective, individualized interventions for neuropsychiatric disorders.

## Data and code availability

This study is a narrative review and did not generate new data or code. All data analyzed in this review were obtained from previously published studies that are publicly available through academic databases. No datasets or software was created by the authors for this work.

## Acknowledgments

This study was supported by the High-Level Talents Introduction Foundation of 10.13039/501100006247Anhui Science and Technology University (DKYJ202402); Veterinary Science Peak Discipline Project of 10.13039/501100006247Anhui Science and Technology University (XK-XJGF002); the University Synergy Innovation Program of Anhui Province (GXXT-2022-076); the Excellent Research Innovation Team in Universities in Anhui Province (2022AH010088). The figures were created with BioRender.com.

## Author contributions

C.J. and W.M.N. conducted the investigation, performed data curation and formal analysis, generated visualizations, and drafted the original manuscript. S.B. and A.H. contributed to the investigation and methodology and participated in manuscript revision and editing. S.M.A. and H.A.A.I. contributed to methodological development and validation and reviewed and edited the manuscript, with H.A.A.I. additionally providing resources. W.Z. and X.Z. performed formal analysis and software-related work and contributed to manuscript revision. C.C. and X.L. participated in investigation and validation and reviewed and edited the manuscript. M.J. contributed to data curation, visualization, and manuscript revision. A.H.G., M.R., and S.L. conceived and supervised the study; A.H.G. and S.L. acquired funding, and M.R. provided additional resources. All senior authors contributed to project administration and manuscript revision. All authors read and approved the final version of the manuscript.

## Declaration of interests

The authors declare no competing interests.
